# Biomass-Derived Carbon Dots as Fluorescent Probes for Label-Free Sensing of Hemin and as Radical Scavengers

**DOI:** 10.3390/bios15020105

**Published:** 2025-02-12

**Authors:** Neha Sharma, Hae-Jeung Lee

**Affiliations:** 1 Department of Food and Nutrition, College of Bionanotechnology, Gachon University, Seongnam-si 13120, Republic of Korea; nehaworld92@gmail.com; 2 Institute for Aging and Clinical Nutrition Research, Gachon University, Seongnam-si 13120, Gyeonggi-do, Republic of Korea; 3 Department of Health Sciences and Technology, Gachon Advanced Institute for Health Science and Technology, Gachon University, Incheon 21999, Republic of Korea

**Keywords:** carbon dots, biomass, antioxidant, fluorescence quenching, sensing, hemin, sensor

## Abstract

Carbon dots (CDs) derived from biomass are promising fluorescent probes for specific analyte detection due to their specificity, biocompatibility, selectivity, and sensitivity. In this work, carbon dots were prepared hydrothermally from natural material, *Myrica esculenta* fruits (hereafter referred to as MPCDs), without adding any chemicals. The prepared MPCDs were characterized using optical, microscopic, and spectroscopic methods that revealed the presence of numerous functional groups and fluorescent properties. MPCDs exhibited exceptional characteristics such as water solubility, photostability, excitation-dependent fluorescence emission, and ionic stability. Transmission electron microscopy found that the average size of the MPCDs was 8 nm. MPCDs exhibited remarkable sensing ability for hemin, with a good linearity (R^2^ = 0.999) and a lower limit of detection of 14.1 nM. MPCDs demonstrated fluorescence quenching-based detection of hemin, primarily owing to ground state complex formation and the inner filter effect. Furthermore, the prepared material exhibited excellent antioxidant potential against 2,2′-azino-bis (3-ethylbenzothiazoline-6-sulfonic acid) and 2,2-diphenyl-1-picrylhydrazyl radicals with EC_50_ values of 25.4 and 205.4 µg/mL, respectively. The study suggests that CDs from *Myrica esculenta* fruits could be used as optical sensors for hemin detection as well as to scavenge selected radicals.

## 1. Introduction

Hemin (Fe^3+^-protoporphyrin IX) is the functional component of myoglobin, hemoglobin, and cytochrome C [1]. It is present in the body of all living organisms. It has a peroxidase-like activity and participates in several cellular functions, such as metabolizing drugs and the transferring of electrons and oxygen [2]. Adequate hemin levels are necessary to maintain normal human health. A deficiency causes oxidative stress and impaired mitochondrial functions, whereas an excess can cause brain hemorrhage, swelling, vasculopathy, and kidney dysfunction [3]. Hemin as a drug has been used widely for treating attacks of acute porphyria, anemia due to iron deficiency, and tumors [4]. The overuse of hemin might result in complications including circulatory failure, vein swelling, and blood clotting disorders [5]. Consequently, the precise detection of hemin is crucial for the early detection of illnesses.

Carbon dots (CDs) are fluorescent nanoparticles exhibiting diameters below 10 nm with excitation-dependent emission properties [6]. The existence of several functional groups, including hydroxyl, carboxyl, and amine, endows them with many qualities such as high-water solubility, extensive absorption range, photostability, biocompatibility, superior electrical characteristics, and non-toxicity [7]. Due to these features, their application in various fields, including sensing, optoelectronics, electrochemistry, bioimaging, drug delivery, biodiesel production, dye degradation, and antioxidation, has been well documented [8,9,10,11,12,13,14,15]. The nature of the precursor material and synthesis process determine the suitability of CDs for any application. Previously, few studies have utilized chemical-based CDs for sensing hemin [16,17,18]. Researchers are increasingly turning their attention from the use of harmful chemicals in favor of more eco-friendly raw materials, such as plants and their components, microorganisms and their waste products, animals, and bio-waste for synthesizing nanomaterials [19]. These materials are easy to obtain, affordable, non-toxic, and found in abundance in nature [20]. Only one research study has reported the use of scallion leaf-derived CDs for the detection of hemin [21].

The subtropical Himalayas are home to diverse popular medicinal plants, including *Myrica esculenta*, a member of the Myricaceae family [22]. The parts of this plant are reported to be used for treating chronic bronchitis, anemia, earache, dysentery, and inflammation [23]. The fruit of this plant offers protection against oxidative stress due to the presence of phenols and flavonoids. Gallic acid, myricetin, phlorizin, ellagic acid, p-coumaric acid, ascorbic acid, cyanin, hydroxybenzoic acid, chlorogenic acid, and catechin are among the many phytoconstituents found in the fruit [24]. A prior study has documented the antioxidant properties of the fruit extracts [25]. Researchers have previously reported the synthesis of metal nanoparticles and non-metal nanoparticles from the extracts of different parts of this plant, like the leaves, bark, and fruit [26,27,28,29,30]. Among these, copper oxide nanoparticles and zinc oxide nanoparticles were reported to be good radical scavengers [27,28]. Despite the nutritional and health benefits of this plant, there are no reports on the use of this plant’s fruit as raw material for CD synthesis.

Hence, this study describes for the first time the synthesis of CDs from the pulp of *Myrica esculenta* fruit (hereafter referred to as MPCDs) utilizing the hydrothermal method. Using a variety of techniques, the MPCDs were characterized to determine their size, shape, chemical composition, and fluorescence properties. Their radical scavenging ability was explored via 2,2′-azino-bis (3-ethylbenzothiazoline-6-sulfonic acid (ABTS) and 2,2 diphenyl-1-picrylhydrazyl (DPPH) antioxidant assays. The study emphasizes the application of MPCDs as a sensitive and precise fluorescent probe for detecting hemin and elucidates the associated sensing mechanisms. The temperature stability, pH stability, and ionic stress tolerance of the constructed sensor were also examined. Moreover, the study illustrated the practical utilization of MPCDs in identifying hemin in biological samples.

## 2. Materials and Methods

### 2.1. Reagents and Chemicals

*Myrica esculenta* fruit was bought from the local market of Mandi, India. Hemin and sodium hydroxide (NaOH) were purchased from Duksan Pure Chemicals, Ansan, South Korea. Ascorbic acid, ABTS, DPPH, quinine sulfate, iron (II) chloride, uric acid, sodium bicarbonate, potassium chloride, and bovine serum albumin were bought from Sigma-Aldrich, Massachusetts, USA. Sodium chloride (NaCl) and hydrochloric acid were purchased from Samchun Chemicals Co., Ltd., Seoul, South Korea. Sulfuric acid, magnesium chloride, and calcium chloride were procured from Daejung Chemicals, Gyeonggi-do, South Korea. All chemicals used were of analytical quality and employed without any additional purification.

### 2.2. Instrumentation

Photoluminescence intensity was recorded using the SCINCO fluoromate FS-2 fluorescence spectrometer (Seoul, South Korea) and absorption spectra were captured using the Cary 300 UV–visible spectrophotometer by Spectralab Scientific Inc. (Ontario, Canada). Transmission electron microscopy (TEM) images of MPCDs were captured using an FEI Titan 80–300 microscope by ThermoFisher Scientific (Massachusetts, USA). The zeta potential was measured using the Nanosizer 500, Anton Paar GmbH (Graz, Austria). A lifetime decay study was carried out using a photo/fluorescence luminescence spectrometer system (HORIBA/Fluorolog-QM), California, USA. The Fourier transform infrared (FTIR) spectra and X-ray photoelectron spectra (XPS) were acquired using a Jasco FT/IR-4600, Tokyo, Japan and ThermoFisher Scientific (Multilab-2000), Massachusetts, USA with Al K X-ray energy source, respectively.

### 2.3. Preparation of MPCDs

The fruit of *Myrica esculenta* were thoroughly washed three times with distilled water. The fruit pulp was extracted carefully from the seeds, mashed in a grinder, and mixed with 100 mL of distilled water by stirring for 30 min. After this, the obtained mixture was placed in the Teflon-lined autoclave vessel for treatment at 178 °C for a duration of 16 h. Following reduction of the autoclave temperature to ambient, the prepared material was taken from the vessel and centrifuged for 10 min at 10,000 rpm to remove large particles and impurities. Finally, the solution was filtered through a 0.22 µm syringe filter and stored in the refrigerator till further use.

### 2.4. Quantum Yield (QY) Measurement

Quinine sulfate was used as a reference material owing to its high QY (54.6%) and similar excitation (350 nm) wavelength to the MPCDs (360 nm). By keeping the absorbance of water-dissolved MPCDs and sulfuric acid-dissolved quinine sulfate below 0.05, the quantum yield of the prepared MPCDs was calculated utilizing the following formula.

QY_MPCD_ = Q_SD_(I_MPCD_/I_SD_)(A_SD_/A_MPCD_)(rf_MPCD_/rf_SD_) (1)

where I stand for fluorescence emission intensity, rf denotes refractive index, and A represents the absorbance. SD in subscript denotes quinine sulfate as standard.

### 2.5. Radical Scavenging Potential of MPCDs

The antioxidant activity of synthesized MPCDs was assessed using DPPH and ABTS assays. Ascorbic acid was used as a standard in both assays. The following equation was utilized for calculating the scavenging percentage of MSTCDs for DPPH and ABTS [31] radicals.

Inhibition % = (A_co_ − A_sp_/A_co)_ × 100 (2)

where A_co_ represents radical absorbance in the absence of MPCDs or control and A_sp_ denotes radical absorbance in the presence of MPCDs or control [32].

#### 2.5.1. DPPH Assay

DPPH assay was performed using a previously reported procedure with a few modifications [32]. A 100 µM methanolic DPPH solution was prepared and mixed with different concentrations (3.9 to 1000 µg/mL) of MPCDs in the ratio of 9:1. The reaction was performed for 15 min in the darkness, and absorbance was recorded at a wavelength of 517 nm using a plate reader (Epoch Biotek, Winooski, VT, USA). Distilled water was used as a blank control, and the assay was performed in triplicate.

#### 2.5.2. ABTS Assay

Using an already documented method [31], the ABTS radical-removing capacity of MPCDs was evaluated. ABTS reagent (7 mM) and potassium persulfate (2.45 mM) mixture in the ratio of 1:0.5 were kept in the dark for 16 h to produce ABTS radicals. After maintaining the absorbance of the ABTS radical solution at 0.7 ± 0.2 at a wavelength of 734 nm, the solution was used for the reaction with different concentrations of the prepared material or standard. A total of 20 µL of sample or standard was mixed with 180 µL of ABTS radicals and incubated for 10 min. The blank control used was distilled water. The results were obtained by taking absorbance at a wavelength of 734 nm. The assay was performed in triplicate.

### 2.6. Detection of Hemin by MPCDs

At room temperature, the sensing of hemin by MPCDs was performed in distilled water at an excitation of 360 nm with a slit width of 5 nm. To evaluate sensitivity for hemin, 2 mL of MPCDs were mixed with different volumes of hemin (1 mM prepared in 0.1 M NaOH) to prepare different concentrations ranging from 0.1 to 26 µM and diluted with distilled water to make the final volume of the reaction mixture 2.5 mL.

### 2.7. Serum Sample Analysis

Commercial serum samples were diluted into a 1:50 dilution with 0.1 M NaOH and then spiked with hemin (1 mM) to make final concentrations of 1, 6, and 12 µM. The fluorescence intensities of the spiked MPCDs were recorded to calculate the recovery percentage. The mean ± standard deviation was used to express data from experiments performed in triplicate.

## 3. Results and Discussion

### 3.1. Synthesis and Optical Characteristics of MPCDs

**Figure 1:**
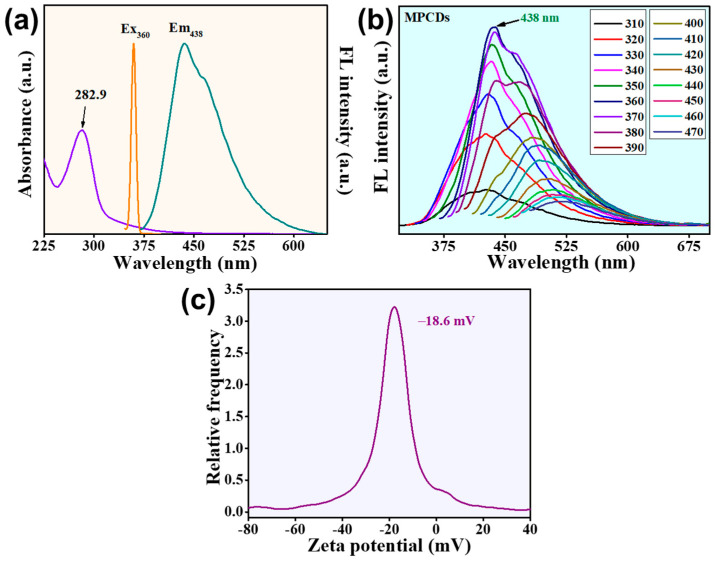
(**a**) UV spectra and maximum excitation and emission spectra of MPCDs. (**b**) Photoluminescence spectra of MPCDs at different excitation wavelengths (310–470 nm). (**c**) Zeta potential of MPCDs.

The synthesis of the MPCDs includes the hydrothermal treatment of *Myrica esculenta* fruit without using any harsh chemicals. As shown in Figure 1a, an absorption band was observed between 245 and 315 nm, depicting the existence of carbonyl groups with a peak centered at 282.9 nm attributed to the π–π* transition of the aromatic ring. The maximum excitation and emission values of the MPCDs were observed to be 360 and 438 nm, respectively, as per the analysis of the fluorescence spectra (Figure 1a).

Figure 1b presents the excitation-dependent emission spectra of MPCDs upon excitation ranging from 310 to 470 nm. This phenomenon can be related to the emissive traps and defects on the surface of the MPCDs. Though the QY of MPCDs was calculated to be low with a value of 3.3%, the zeta potential of the prepared material predicts its colloidal stability. In this study, the zeta potential of the prepared material was found to be −18.6 mV (Figure 1c), which infers that the surface of the MPCDs carries an electro-negative charge, bestowing them with stability in liquid solutions [33].

### 3.2. Morphological Characteristics of MPCDs

**Figure 2:**
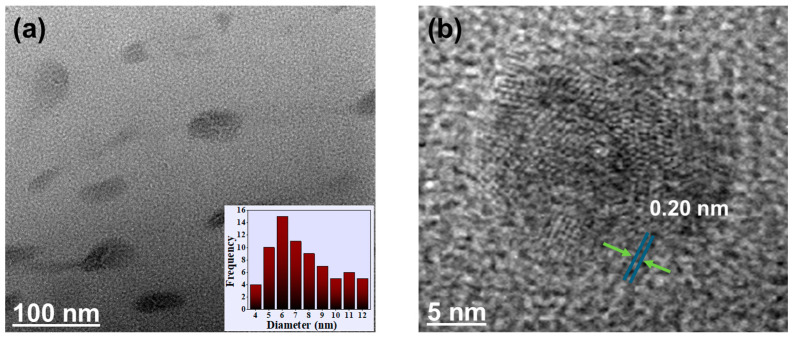
(**a**) Transmission electron microscopic (TEM) image and distribution of particle size (inset) of MPCDs. (**b**) High-resolution TEM image of MPCDs.

To explore the dimensions of the MPCDs, TEM analysis was performed. The TEM images in Figure 2a show that the as-prepared MPCDs were uniformly dispersed. The size distribution ranged from 4–12 nm, and the average size from the size distribution histogram was calculated to be 8 nm (inset in Figure 2a). The MPCDs possessed a semi-spherical shape with a lattice spacing of 0.20 nm (Figure 2b).

### 3.3. Elemental Characteristics of MPCDs

**Figure 3:**
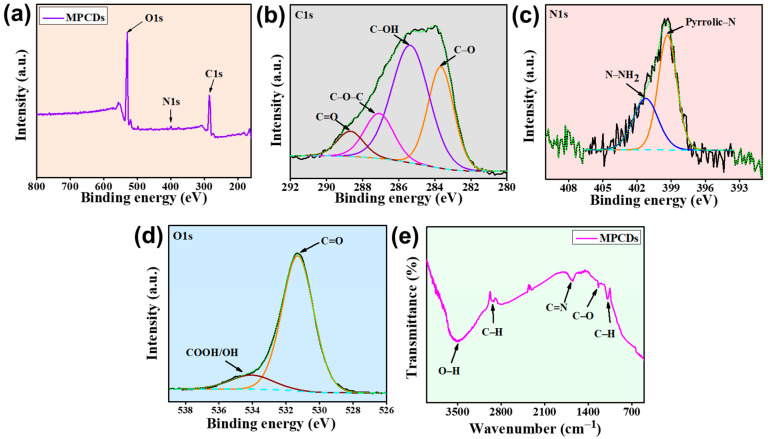
(**a**) XPS survey scan of MPCDs with corresponding deconvoluted spectra of (**b**) C1s, (**c**) N1s, and (**d**) O1s. (**e**) FTIR spectra of MPCDs.

The elemental makeup and functional groups of the MPCDs were explored using XPS and FTIR analysis. The presence of various chemical groups determines the usability of CDs for various applications. The XPS survey scan of the MPCDs confirms the presence of elements carbon (C1s), nitrogen (N1s), and oxygen (O1s) with their corresponding peaks at 284.6, 399.3, and 529.8 eV, respectively (Figure 3a). The four peaks at 288.6, 287.1, 285.4, and 283.6 eV in the high-resolution C1s spectra (Figure 3b) could be recognized as C=O, C-O-C, C-OH (hydroxyl), and C-O bond, respectively. The N1s spectra of MPCDs can be divided into two peaks positioned at 401.2 and 399.3 eV, corresponding to amino (N-NH_2_) and pyrrolic nitrogen (pyrrolic-N) (Figure 3c). Furthermore, the binding energies observed at approximately 534 and 531.3 eV in the deconvoluted high-resolution spectra of O 1s were assigned to COOH/OH and C=O (sp^2^) functional groups, respectively (Figure 3d) [34].

The stability, dispersibility, and hydrophilicity of CDs are enhanced by the functional groups on their surface [35]. The functional groups of MPCDs were further identified using FTIR spectroscopy. As shown in Figure 3e, the broad band centered at 3478 cm^−1^ was related to the stretching vibrations of O-H, and the band at 2930.1 cm^−1^ was attributed to C-H stretching vibrations [36]. The absorption bands at 1654 and 1234 cm^−1^ were due to C=N and C-O stretching, respectively [37]. The presence of aromatic C-H stretch was indicated by the peak at 1088 cm^−1^. As indicated by the surface analysis results, the FTIR data validate that MPCDs are functionalized with amine (C–NH_2_), carboxyl (C=O), and hydroxyl (C-OH) groups.

### 3.4. Stability of MPCDs

**Figure 4:**
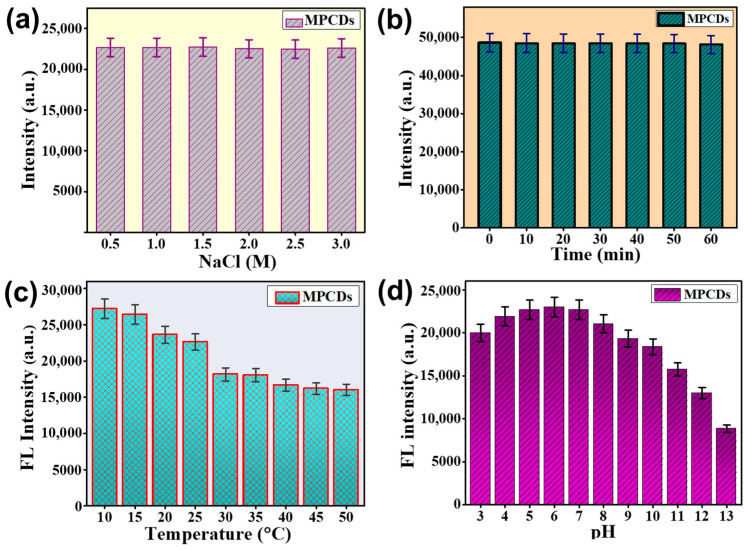
The fluorescence intensity of MPCDs in different conditions: (**a**) in NaCl ionic solutions (0.5–3 M), (**b**) at 365 nm UV irradiation (0–60 min), (**c**) at temperatures varying from 10 to 50 °C, and (**d**) in different pH buffers (3–13). The error bars show the standard deviations calculated from three separate experiments.

The investigation of the stability of the MPCDs under various parameters was performed. The storage stability of the MPCDs is shown in Figure S1. It was observed that the fluorescence intensity of the MPCDs remained consistent throughout the four months of storage, suggesting their good storage stability. The stability of MPCDs under ionic stress conditions was studied using different molar solutions of NaCl. As depicted in Figure 4a, no effect on the fluorescence intensity of the MPCDs was observed when the ionic strengths were varied from 0.5 to 3 M. A good ionic salt tolerance was displayed by MPCDs, which shows their usability for practical applications [38]. Further, as shown in Figure 4b, the MPCDs showed a significant ability to withstand ultraviolet irradiation (at 365 nm) when exposed for 60 min. In Figure 4c, the effect of different temperature treatments on the fluorescence intensity of MPCDs is shown. It was observed that at a lower temperature of 10 °C, the highest fluorescence intensity was obtained. With an increase in temperature from 10 °C to 35 °C, a decrease in the fluorescence intensity was witnessed, and at higher temperatures after 40 °C, no significant change in the intensity was recorded. Therefore, the experiments were conducted at an optimal temperature. In addition to temperature, the effect of pH change on the fluorescence intensity of the MPCDs was also studied. As shown in Figure 4d, the fluorescence intensity of MPCDs was enhanced with a rise in pH from 3 to 5, and from pH 5 to 7, the fluorescence intensity was observed to be the same. A further increase in the pH resulted in a decrease in the fluorescence intensity of the MPCDs. A change in the pH of the medium causes a photobleaching reaction that lowers the fluorescence [39]. Since the original pH of the prepared MPCDs was 5, the sensing experiments were performed using MPCDs without any further pH modification.

### 3.5. Antioxidant Activity

The ability of MPCDs to scavenge radicals was evaluated using two widely employed chemical-based methods, namely DPPH and ABTS.

#### 3.5.1. DPPH

**Figure 5:**
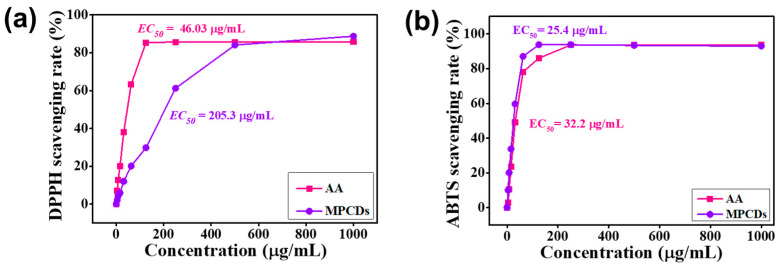
Antioxidant potential of MPCDs against (**a**) DPPH. (**b**) ABTS radicals. DPPH: 2, 2 diphenyl 1 picrylhydrazyl, AA: ascorbic acid, ABTS: 2,2′-azino-bis (3-ethylbenzothiazoline-6-sulfonic acid.

DPPH is a well-known, straightforward, and dependable test for determining a compound’s radical scavenging activity. DPPH is a persistent free radical that contains nitrogen and produces a stable DPPH2 complex when it comes into contact with a hydrogen radical. Figure 5a illustrates that the scavenging capacity of the MPCDs and ascorbic acid increased with rising concentrations. The maximum scavenging of 88.7% at 1000 µg/mL was achieved by the MPCDs. The EC_50_ (effective concentration of the substance that elicits a half-maximal response) values of the MPCDs and ascorbic acid were determined to be 205.3 and 46.03 µg/mL, respectively. As described above, the functional groups, such as carboxylic, amino, and hydroxyl, present over the surface of MPCDs could have functioned as hydrogen donors, resulting in DPPH scavenging [40]. The favorable antioxidant activity of the MPCDs demonstrates their efficacy as an antioxidant agent.

#### 3.5.2. ABTS

The neutralization of ABTS^•+^ radicals entails the uptake of electrons from antioxidants. MPCDs efficiently scavenged the ABTS^•+^ radicals as depicted in Figure 5b. As the concentration of the MPCDs was enhanced from 3.9 to 125 µg/mL, their scavenging capacity also rose from 10.1% to 93.7%. The MPCDs could scavenge up to 93% ABTS^•+^ at 250 µg/mL. The scavenging potential remained unchanged upon the addition of MPCDs at higher concentrations. Interestingly, the EC_50_ value of the MPCDs (25.4 µg/mL) was lower than that of ascorbic acid (32.2 µg/mL), suggesting that MPCDs have a higher antioxidant potential against ABTS radicals. It has been reported that the presence of various functional groups on the surface of CDs facilitates the elimination of radicals [41]. This suggests that MPCDs could be used as effective antioxidant material; however, antioxidant properties need to be evaluated in food products, and compatibility studies should be evaluated in cell and animal models.

### 3.6. Hemin Detection by MPCDs

**Figure 6:**
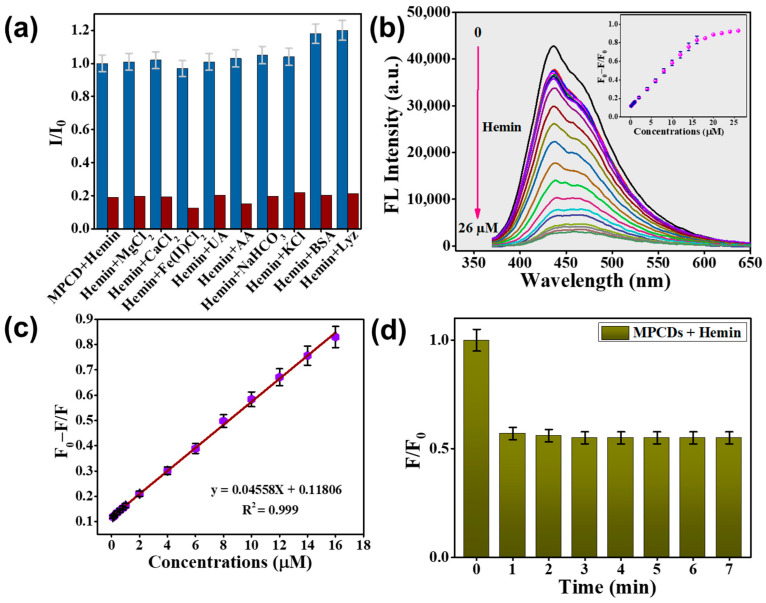
(**a**) Selectivity study of MPCDs for hemin (20 µM) detection in the presence of various interferents at 200 µM concentration. (**b**) Decrease in the fluorescence of MPCDs after addition of hemin at concentrations from 0 to 26 µM. (**c**) Line graph showing the correlation between the fluorescence intensity ratio (F_0_–F/F) of MPCDs and different concentrations (µM) of hemin. (**d**) Equilibrium time study for the detection of hemin by MPCDs. The error bars represent the standard deviations calculated from three separate experiments. MgCl_2_: Magnesium chloride, CaCl_2_: Calcium chloride, Fe(II)Cl_2_: Iron(II) chloride, UA: Uric acid, AA: Ascorbic acid, NaHCO_3_: Sodium bicarbonate, KCl: Potassium chloride, BSA: Bovine serum albumin, Lyz: Lysozyme.

Before performing a hemin detection analysis, we evaluated the selectivity of the MPCDs towards hemin, taking into account the intricate nature of actual samples. Figure 6a illustrates that the fluorescence intensity of the MPCDs remained almost unchanged with the addition of interferents at a concentration of 200 µM.

It is interesting to note that lysozyme and bovine serum albumin increased the fluorescence intensity of the MPCDs when hemin was absent, but they had no effect on the MPCDs’ ability to sense hemin when they were present together. Hemin (at a concentration of 20 µM) efficiently quenched the fluorescence intensity of the MPCDs in the presence of interferents including ions, metals, and other biomolecules. The MPCDs demonstrated a remarkable ability to selectively detect hemin.

Following the validation of the selectivity of the MPCDs for hemin, a stability study of MPCDs for sensing hemin (10 µM) was performed at temperatures ranging from 10 to 50 °C. Interestingly, at the lower temperature of 10 °C, the fluorescence intensity was higher compared to temperatures from 15 to 50 °C (Figure S2). Different temperatures showed minimal effect on the MPCDs’ sensing ability for hemin, indicating the excellent thermal stability of the MPCDs. The effect of storage for 3 months on the hemin sensing capability of MPCDs was also assessed. Figure S3 illustrates the negligible variation in the fluorescence intensity of the MPCDs even after storing for 3 months.

Furthermore, the detection capability of the MPCDs was investigated by assessing the variation in fluorescence intensity with the addition of increasing concentrations of hemin (0–26 µM). As depicted in Figure 6b, with an increase in the hemin content, a gradual decrease in the fluorescence intensity of the MPCDs at an emission wavelength of 438 nm was observed. From the plot of F_0_ − F/F_0_ shown in Figure 6c, good linearity was found in the range of 0.1 to 16 µM of hemin, and the equation is expressed as y = 0.04558x + 0.11806 with a correlation coefficient (R^2^) of 0.999. The limit of detection (LOD) was calculated to be 14.1 nM using the standard formula, LOD = 3sd/s, where sd is the standard deviation and s is the slope [42]. Of note, the calculated LOD in this study was found to be lower than the LODs reported from previously documented studies on CDs prepared from green sources (Table 1).

With the exception of one study that used the microwave synthesis method [18], all previously published CDs were created using the hydrothermal process. The o-phenylenediamine-derived CDs were synthesized at 150 °C for 8 h. The resulting CDs exhibited red fluorescence and detected hemin via the inner filter effect (IFE) and fluorescence resonance energy transfer [16]. N,Cl-doped CDs [17] and N,S-doped CDs [21] were synthesized at 190 °C for 6 h and 180 °C for 12 h, respectively. Hemin detection using N,Cl-doped CDs was attributed to the IFE, whereas N,S-doped CDs exhibited quenching effects through both IFE and photo-induced electron transfer mechanisms [17,21]. N,Cl-doped CDs also exhibited temperature-dependent sensing [17]. CDs synthesized from citric acid, magnesium chloride, and ethylenediamine precursors were prepared via a microwave method (2 min treatment) and detected hemin via IFE [18]. Our study showed significant differences compared to these studies (Table 1): (1) lower LOD, (2) utilization of sustainable material without chemical pretreatment, (3) absence of doped elements, (4) hemin detection via static quenching and IFE, and (5) reasonably good radical scavenging activity. However, the chemical-based magnesium-doped CDs showed a lower LOD (1 nM) than our MPCDs. This could be due to magnesium doping in the reported CDs. Furthermore, except for one study [21], all the studies described the use of chemicals for the preparation of CDs for hemin detection. This indicates that the CDs reported in our study and by Zhang et al. [21] demonstrated a cost-effective synthesis method for hemin detection. Although electricity consumption appears lower in microwave production, the use of expensive chemicals renders the detection approach more costly. This suggests that the physiochemical and optical properties of CDs depend upon the precursor material and synthesis reaction conditions. Investigations were also carried out into the duration of time needed for the optimum reaction between MPCDs and hemin. Figure 6d depicts the F/F_0_ (F and F_0_ are the intensity of MPCDs in the presence and absence of hemin, respectively) values of MPCDs plotted against different times (0–7 min). The addition of hemin quenched the fluorescence intensity of the MPCDs from 1 to 3 min. After 3 min of reaction, no noticeable change in the fluorescence intensity of the MPCDs was observed, suggesting that it takes 3 min for MPCDs and hemin to reach equilibrium. The results indicate the MPCD probe is a fast and stable sensor for detecting hemin. Despite the remarkable hemin sensing ability and effective radical scavenging activity, this study also has limitations. The study has not conducted a biocompatibility analysis of the prepared CDs, which is crucial for therapeutic applications. Another limitation is the batch-to-batch variability of the prepared CDs, which can influence the physicochemical and optical properties of the material produced.

### 3.7. Possible Mechanisms of Hemin Sensing by MPCDs

**Figure 7:**
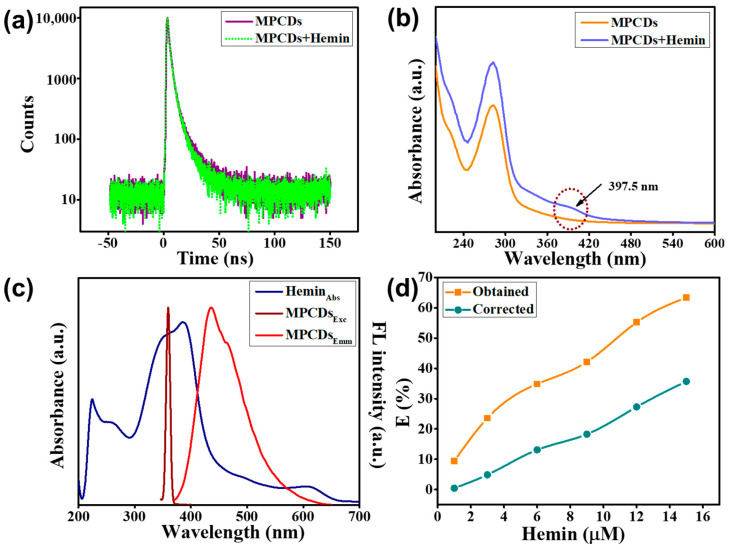
(**a**) Lifetime decay curves of MPCDs without and with hemin. (**b**) UV peaks of MPCDs and MPCDs with hemin. (**c**) UV peaks of hemin with excitation and emission peaks of MPCDs. (**d**) The suppressed efficiency (E%) of obtained and corrected fluorescence intensity for the MPCDs following the addition of various doses of hemin.

The fluorescence quenching phenomenon responsible for the detection of hemin by MPCDs was further investigated using various optical experiments such as fluorescence lifetime decay, UV spectra, and fluorescence spectra. Figure 7a displays the lifetime decay curves of MPCDs without and with hemin.

Their respective decay times were found to be 2.4 and 2.23 ns, exhibiting no significant change, indicating that quenching occurred due to a static quenching mechanism. The UV spectra of the MPCDs and hemin combined with the MPCDs were studied to further understand the detection mechanism. Figure 7b reveals the appearance of a new peak at 397.5 nm corresponding to the n-π transition of C=O bonds in the combined UV spectra of the MPCDs and hemin, implying the formation of a complex between the MPCDs and hemin. In addition, UV spectra of hemin and excitation and emission fluorescence spectra of the MPCDs were plotted together to explore their overlapping pattern (Figure 7c). The excitation peak of the MPCDs overlaps with the UV peak of hemin, inferring the involvement of IFE in the quenching [43].

The measured fluorescence emission intensity of the MPCDs was adjusted using the Parker equation to validate the existence of an IFE [44].

(3) $$\frac{F_{cor}}{F_{ob}}=\frac{2.3dA_{ex}}{{1-10}^{-dA_{ex}}}10^{gA_{em}}\frac{2.3sA_{em}}{1-10^{-sA_{em}}}$$

where F_ob_ is the obtained fluorescence intensity and F_cor_ is the IFE-corrected fluorescence intensity of the MPCDs. A_em_ is the absorbance at the 438 nm emission wavelength, while A_ex_ is the absorbance at the 360 nm excitation wavelength. g represents the length between the edges of the cuvette and the excitation beam. The width of the cuvette is 1.0 cm, denoted as d. s is the excitation beam’s thickness, which is 0.10 cm. Equation (3) was used to compute the correction factor (CF) and obtained (E_ob_), and IFE-corrected (E_cor_) fluorescence quenching efficiencies of the MPCDs. The results are compiled in Table 2. As shown in Figure 7d, 36% of the quenching of fluorescence was due to IFE, which suggests that IFE also contributed to the quenching process of the MPCDs in the presence of hemin.

Further, Figure 7c displays that the emission spectra of the MPCDs exhibit local overlap with the UV peak of hemin, suggesting that the cause of quenching was also Förster resonance energy transfer (FRET). Thus, the above results show that hemin not only formed a ground complex with the MPCDs but also absorbed energy in the ground as well as in the excited state [45]. The prepared MPCDs displayed a remarkable hemin sensing ability and good DPPH and ABTS radical scavenging ability. Both these properties could be due to the interactive transfer of electrons and hydrogen from the surface functional groups of the MPCDs, which include carboxyl (−COOH), hydroxyl (−OH), and amino (−NH_2_), to radicals and hemin.

### 3.8. Detection of Hemin in Commercial Serum Samples by MPCDs

To ensure the constructed fluorescence-detecting probe was suitable for practical use, it was tested for detecting hemin levels in commercial serum samples. The recovery experiment was performed by adding different amounts of hemin to the MPCDs. The results are summarized in Table 3. The recoveries ranged from 98.7 to 107.3% and the relative standard deviation varied from 1.5 to 1.9%. The results showed that this probe could be used as a substitute with excellent precision and repeatability in serum samples for hemin detection.

## 4. Conclusions

This study reports for the first time the synthesis of CDs using fruits of *Myrica esculenta* based on the principle of green chemistry through a hydrothermal approach. The size of the as-prepared MPCDs was demonstrated to be in the range of 4–12 nm. Due to high water solubility, the prepared MPCDs demonstrated exceptional sensing ability for hemin based on static quenching and IFE. Importantly, the developed sensor showed a lower LOD (14.1 nM) compared to the previously reported studies on green CDs for hemin detection. Furthermore, the prepared material exhibited excellent antioxidant potential against DPPH and ABTS radicals. In conclusion, the developed material proved to be an excellent optical sensor for hemin detection. Further studies are needed to evaluate its antioxidant ability in cell and animal models.

## Figures and Tables

**Table 1:** A comparison of fluorescence-based methods utilizing CDs as a probe for the sensing of hemin.

Source	Probe	Method	Linear Range (µM)	LOD (nM)	Year	Reference
o-phenylenediamine	N-doped CDs	Hydrothermal	0.4–32	180	2019	[16]
Hexadecylpyridinium chloride	N,Cl-doped CDs	Hydrothermal	1–32	160	2020	[17]
Citric acid, magnesium chloride, and ethylenediamine	Mg-CDs-0.1 Mg-CDs-0.5	Microwave	0.1–75 0.01–25	25 1	2020	[18]
Scallion leaves	N,S-doped CDs	Hydrothermal	0.5–10	100	2020	[21]
*Myrica esculenta* fruit	MPCDs	Hydrothermal	0.1–16	14.1	-	This work

CDs: carbon dots, LOD: limit of detection, N: nitrogen, Cl: chlorine, Mg: magnesium, S: sulfur.

**Table 2:** Inner filter effect of hemin on the fluorescence of MPCDs.

Hemin (μM)	A_exc_	A_emm_	F_obs_	F_cor_	CF F_cor_/F_obs_	F_cor,blank_/F_cor_	E_obs_ %	E_cor_ %
0	0.291	0.116	34,542	53,325	1.54	1	0	0
1	0.359	0.141	31,278	53,076	1.72	1.00	9.44	0.46
3	0.454	0.172	26,388	50,722	1.92	1.05	23.60	4.88
6	0.491	0.205	22,498	46,343	2.05	1.15	34.86	13.09
9	0.531	0.227	19,985	43,712	2.18	1.21	42.14	18.28
12	0.627	0.275	15,469	38,777	2.50	1.37	55.21	27.28
15	0.664	0.319	12,649	34,278	2.71	1.55	63.38	35.71

**Table 3:** Determination of hemin in commercial serum samples by MPCDs (*n* = 3).

Samples	Spiked (µM)	Found	Recovery (%)	RSD (%)
1	1	1.07	107.3	1.5
2	8	7.89	98.7	1.9
3	14	14.06	100.4	1.7

RSD: relative standard deviation.

## Data Availability

Dataset available on request from the authors.

## Supplementary Materials

The following supporting information can be downloaded at: https://www.mdpi.com/article/10.3390/bios15020105/s1, Figure S1: Storage stability of MPCDs. Figure S2: Effect of temperature (10-–50 °C) on the sensing ability of MPCDs for hemin (10 μM). Figure S3: Stability study of stored MPCDs for detecting hemin.
