# Supplementary material for: Biomass-Derived Carbon Dots as Fluorescent Probes for Label-Free Sensing of Hemin and as Radical Scavengers
Source: Biosensors (Basel). 2025 Feb 12;15(2):105. doi: 10.3390/bios15020105 (PMC11853633; doi:10.3390/bios15020105)
Supplement: Supplementary file 1 [file biosensors-15-00105-s001.zip › biosensors-3436429-supplementary.pdf]

biosensors

Article

Biomass-Derived Carbon Dots as Fluorescent Probes for Label-Free Sensing of Hemin and as Radical Scavengers

Neha Sharma [1] and Hae-Jeung Lee [1,2,3,*]

[1] Department of Food and Nutrition, College of Bionanotechnology, Gachon University, Seongnam-si 13120, Republic of Korea; nehaworld92@gmail.com
[2] Institute for Aging and Clinical Nutrition Research, Gachon University, Seongnam-si 13120, Gyeonggi-do, Republic of Korea
[3] Department of Health Sciences and Technology, Gachon Advanced Institute for Health Science and Technology, Gachon University, Incheon 21999, Republic of Korea
* Correspondence: skysea@gachon.ac.kr or skysea1010@gmail.com; Tel.: +82-31-750-5968; Fax: +82-31-750-5974

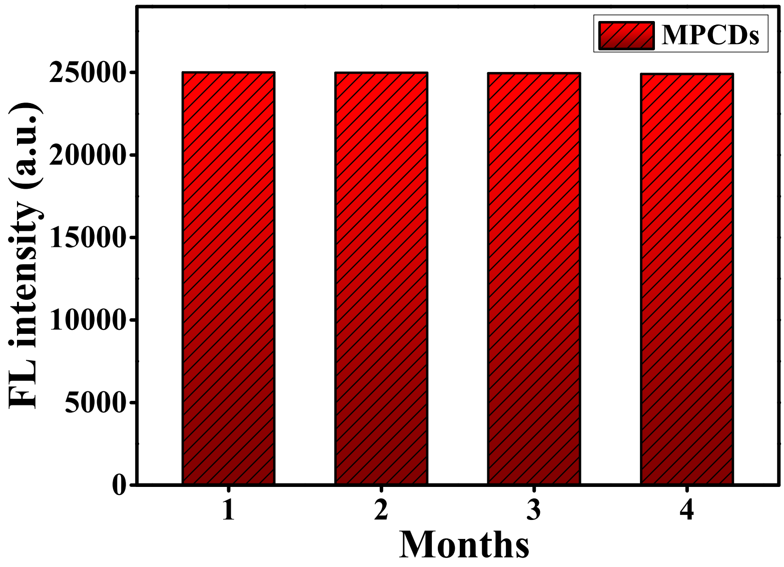

Fig. S1. Storage stability of MPCDs.

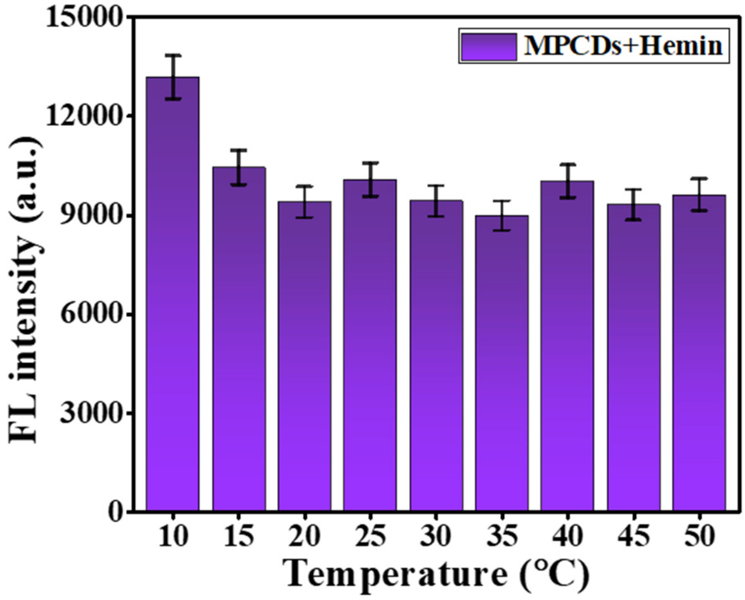

Fig. S2. Effect of temperature (10-50 °C) on the sensing ability of MPCDs for hemin (10 μM).

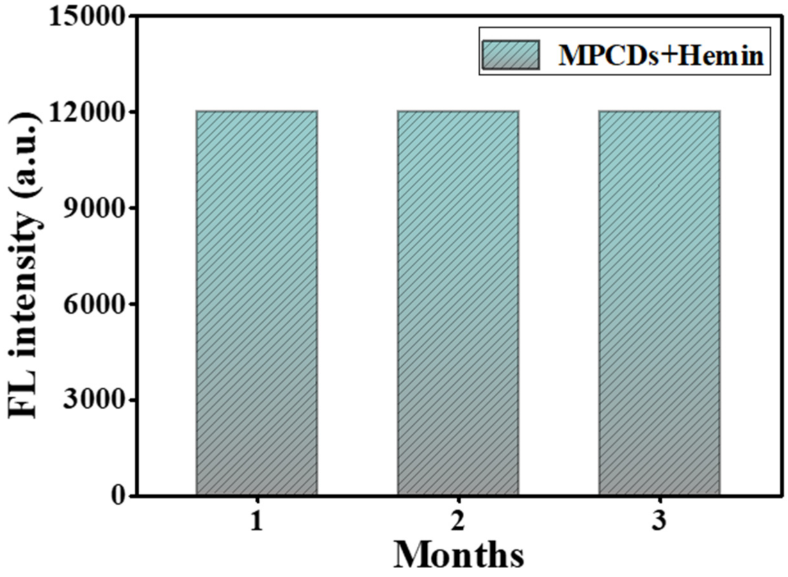

Fig. S3. Stability study of stored MPCDs for detecting hemin.
